# NEDDylation of PB2 Reduces Its Stability and Blocks the Replication of Influenza A Virus

**DOI:** 10.1038/srep43691

**Published:** 2017-03-02

**Authors:** Tinghong Zhang, Zhen Ye, Xiaohai Yang, Yujie Qin, Yi Hu, Xiaomei Tong, Wenbin Lai, Xin Ye

**Affiliations:** 1 CAS Key Laboratory of Pathogenic Microbiology and Immunology, Institute of Microbiology, Chinese Academy of Sciences (CAS), Beijing, China; 2 Graduate University of Chinese Academy of Sciences, Beijing, China; 3 School of Life Sciences, Anhui University, Hefei, China; 4 Institute of Health Sciences, Anhui University, Hefei, China; 5 Savaid Medical School, University of Chinese Academy of Sciences, Beijing, China.

## Abstract

Post-translational modifications of viral proteins play important roles in regulating viral replication. Here we demonstrated that the PB2 of influenza A virus (IAV) can be modified by NEDD8. We revealed that E3 ligase HDM2 can promote PB2 NEDDylation. Overexpression of either NEDD8 or HDM2 can inhibit IAV replication, while knockdown of HDM2 has the opposite effect. Then we identified residue K699 in PB2 as the major NEDDylation site. We found that NEDDylation deficient PB2 mutant (PB2 K699R) has a longer half-life than wild-type PB2, indicating that NEDDylation of PB2 reduces its stability. We generated an IAV mutant in which PB2 was mutated to PB2 K699R (WSN-PB2 K699R) and examined the replication of WSN and WSN-PB2 K699R viruses in both MDCK and A549 cells and found that the replication of WSN-PB2 K699R was more efficient than wild-type WSN. In addition, we observed that overexpression of NEDD8 significantly inhibited the replication of WSN, but not WSN-PB2 K699R. The infection assay in mice showed that WSN-PB2 K699R exhibited enhanced virulence in mice compared to WSN, suggesting that NEDDylation of PB2 reduced IAV replication *in vivo*. In conclusion, we demonstrated that NEDDylation of PB2 by HDM2 negatively regulates IAV infection.

Influenza A virus (IAV) is a genetically diverse pathogen responsible for seasonal epidemics as well as pandemics in humans^1^,^2^,^3^. IAV belongs to the family *Orthomyxoviridae*, and is an enveloped virus encoding up to 16 proteins^4^,^5^.

The IAV genome consists of eight segments of negative sense, single stranded RNA, each of which is packaged into separate ribonucleoprotein (RNP) complexes containing RNA polymerase and nucleoprotein (NP)^4^,^6^,^7^,^8^. The transcription and replication of IAV are carried out by the viral RNA-dependent RNA polymerase (RdRp). RdRp contains three subunits: polymerase basic protein 1 (PB1), polymerase basic protein 2 (PB2) and polymerase acidic protein (PA). PB1 contains the polymerase active site which can catalyze the addition of nucleotides. PB2 is responsible for binding to the 5΄ cap of nascent host pre-mRNAs to facilitate cleavage by PA into short capped RNA fragments, which are used as primers for viral transcription^9^,^10^. Meanwhile, PB2 has been implicated in virulence and host adaptation. It has been reported that PB2 can interact with mitochondrial antiviral signaling protein (MAVS) and inhibit IFN-beta induction^11^. Several residues in PB2 have been found to be important for host adaptation^12^,^13^,^14^,^15^.

IAV relies on host proteins and machinery to complete its life cycle. Ubiquitin and ubiquitin-like systems play pivotal roles in the replication of IAV. For example, the nucleoprotein (NP) of IAV utilizes the host ubiquitin system to increase its RNA-binding affinity, in order to promote viral genome replication^16^. SUMOylation of IAV M1 facilitates the nuclear export of vRNP, which is benefit for viral assembly and morphogenesis^17^.

As a ubiquitin-like protein, NEDD8 (neural precursor cell expressed developmentally down-regulated 8) is attached to its substrates during NEDDylation. This process is similar to ubiquitination^18^ and SUMOylation^19^,^20^. First, NEDD8 is activated by a NEDD8-activating enzyme (APPBP1/Uba3 heterodimer). NEDD8 is then transferred to E2-conjugating enzyme, Ubc12/UBE2M or UBE2F. Finally, E3 ligase will transfer NEDD8 from E2 to its target protein^21^,^22^. NEDDylation can be reversed by two deneddylases (NEDP1 and CSN5), which catalyze the deconjugation of NEDD8 from its substrates^23^,^24^. NEDDylation is now accepted widely to be an important modification mechanism to regulate protein activity, stability, cellular localization, and protein–protein interactions. Furthermore, it has been shown that NEDDylation plays important roles during the life cycle of several viruses, and several viral proteins were found to be NEDDylated^25^. For human immunodeficiency virus type-1 (HIV-1), NEDD8 acts through p27 and β-catenin pathways to disrupt adipogenesis and consequent lipodystrophy in patients affected by HIV infection under HAART therapy^26^,^27^. For Kaposi’s sarcoma-associated herpesvirus, NEDDylation is essential for viral latency because it is critical for the viability of KSHV infected lymphoma cells and for the replication of viral genome^28^. However, the relevance of NEDDylation modification with IAV is not well understood.

In this study, we demonstrate that the PB2 of IAV can be NEDDylated at residue K669 by HDM2 during infection. NEDDylation of PB2 reduces its stability and in turn inhibits viral replication, whereas mutation of the PB2 NEDDylation site favors viral replication and enhances virulence in mice. We provide evidence that NEDDylation modification plays an important role in regulating the replication of IAV.

## Results

### PB2 is conjugated to NEDD8

**Figure 1:**
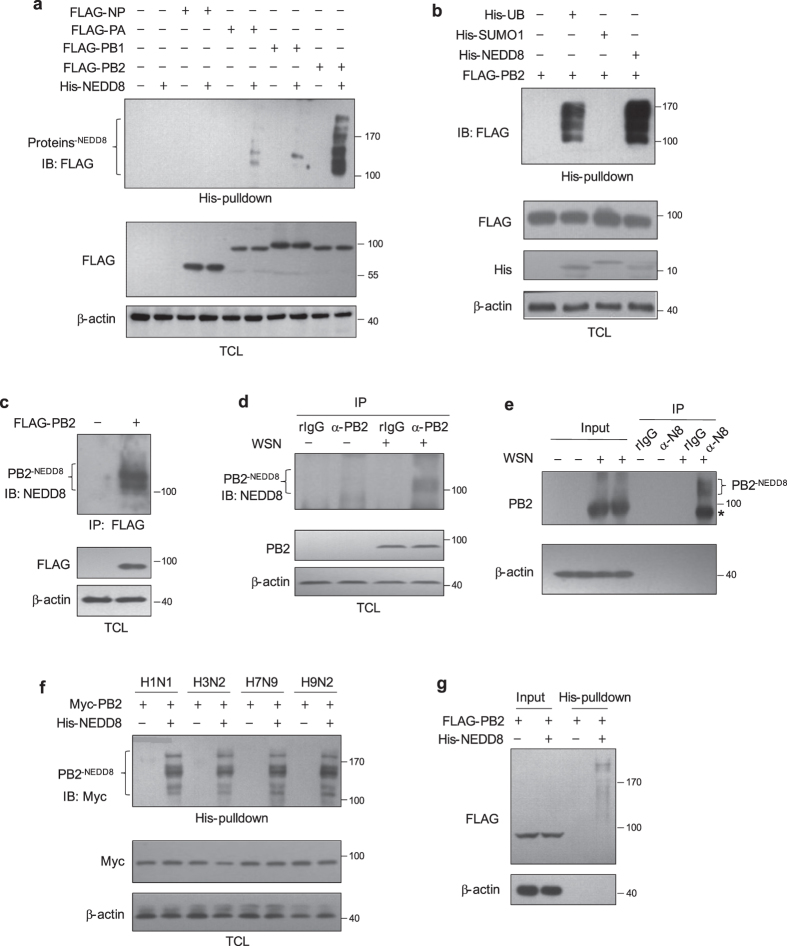
PB2 can be NEDDylated. (**a**) HEK293T cells were transfected with plasmids encoding FLAG-NP, FLAG-PA, FLAG-PB2, or FLAG-PB1 with or without pEF-His-NEDD8. (**b**) HEK293T cells were transfected with FLAG-PB2 together with His-ubiquitin, His-SUMO1 or His-NEDD8, respectively. For (**a**) and (**b**), the cell lysates were harvested for His-pulldown assay followed by immunoblotting with anti-FLAG antibody. (**c**) HEK293T cells were transfected with pCMV FLAG-PB2 or empty vector as control. The cell lysates were subjected to immunoprecipitation with anti-FLAG agarose beads, and immunoblotted with anti-NEDD8 antibody. (**d**) A549 cells were infected with WSN virus at an MOI of 0.1 for 16 h. The cell lysates were subjected to immunoprecipitation with anti-PB2 antibody or rabbit IgG as negative control, and immunoblotted with anti-NEDD8 antibody. (**e**) A549 cells were infected with WSN virus at an MOI of 0.1 for 16 h. The cell lysates were subjected to immunoprecipitation with anti-NEDD8 antibody or rabbit IgG as negative control, and immunoblotted with anti-PB2 antibody. 1/20 of cell lysates was used as the input. The intensity of NEDDylated PB2 and input PB2 were measured by Quantity One. (**f**) pCMV myc-PB2 from 4 different IAV subtypes (A/WSN/1933 (H1N1); A/Guangdong/ST798/2008 (H3N2); A/Anhui/1/2013 (H7N9) and A/HK/2108/2003 (H9N2)) were separately co-transfected with or without pEF-His-NEDD8 into HEK293T cells. After 48 h, cell lysates were harvested for His-pulldown assay followed by immunoblotting with anti-Myc antibody. TCL: total cell lysate. (**g**) HEK293T cells were transfected with FLAG-PB2 together with or without His-NEDD8, and cell lysates were collected for His-pulldown assay. 1/10 of total cell lysates was used as input. The intensity of NEDDylated PB2 and input PB2 were measured by Quantity One. *Unmodified PB2 non-specifically pulled-down by anti-NEDD8 antibody.

In recent years several ubiquitin-like (UBL) proteins, such as SUMO1 and ISG15, have been identified to regulate IAV replication. In order to determine whether vRNP proteins can be modified by NEDD8, we transfected HEK293T cells with FLAG-tagged PB1, PB2, PA or NP expression plasmids together with His-NEDD8 expression plasmid, and then performed the His-pulldown assay. As shown in Fig. 1a, NEDDylated protein bands can be observed in cells expressing FLAG-PB2 and His-NEDD8, a few weak bands were seen from cells expressing PA and PB1 with His-NEDD8 and there was no band in NP expressed cells, indicating that PB2 is obviously modified by NEDD8. Interestingly, we confirmed that PB2 can also be ubiquitinated, which is consistent with previous report^29^ (Fig. 1b). However, we did not observe that PB2 can be modified by SUMO1.

We then examined whether PB2 can be modified by endogenous NEDD8. HEK293T cells were transfected with FLAG-PB2 expression plasmid and the cell lysates were subjected to immunoprecipitation with FLAG antibody, followed by immunoblotting with NEDD8 antibody. PB2 can also be NEDDylated by endogenous NEDD8 (Fig. 1c).

Next, we investigated whether PB2 was NEDDylated during IAV infection. We infected A549 cells with the A/WSN/33 (H1N1; WSN) virus and harvested the cell lysates for immunoprecipitation with PB2 antibody, followed by immunoblotting with NEDD8 antibody. The data showed that NEDDylated PB2 can be detected in virus infected cells (Fig. 1d), indicating that viral PB2 can be modified by NEDD8.

To quantify the ratio of NEDDylated PB2 in infected cells, A549 cells were infected with WSN virus. Then the cells were subjected to immunoprecipitation with NEDD8 antibody with 1/20 of total cell lysates as input, followed by immunoblotting with PB2 antibody (Fig. 1e). The intensity of NEDDylated PB2 and input PB2 was quantified. The ratio of NEDDylated PB2 was approximately 3.9% of total PB2.

We next examined if PB2 proteins derived from other IAV strains can also be modified by NEDD8. Expression plasmids for PB2 from four IAV subtypes (A/WSN/1933 (H1N1); A/Guangdong/ST798/2008 (H3N2); A/Anhui/1/2013 (H7N9); and A/HK/2108/2003 (H9N2)) were cotransfected with pEF-His-NEDD8 into HEK293T cells, and cell lysates were subjected to His-pulldown assay as described previously. The data demonstrated that PB2 from these IAV strains can also be NEDDylated (Fig. 1f).

To quantify the ratio of NEDDylated versus total PB2 in transfected cells, HEK293T cells were transfected with FLAG-PB2 along with or without His-NEDD8. Cell lysates were then subjected to His-pulldown assay with 1/10 of lysates as input (Fig. 1g). The intensity of NEDDylated and input PB2 was quantified, and the ratio of NEDDylated PB2 was determined to be approximately 9.3% of total PB2.

### HDM2 is the NEDDylation E3 ligase for PB2

**Figure 2:**
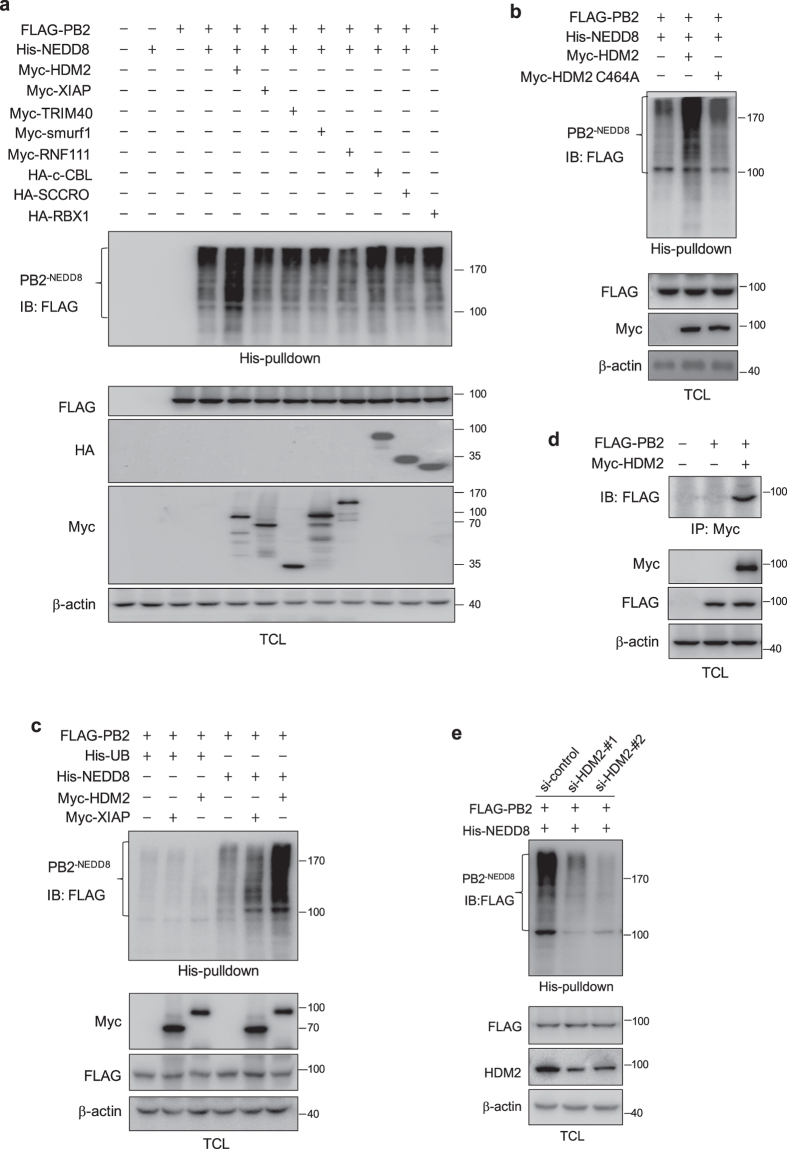
E3 ligase HDM2 promotes PB2 NEDDylation, whereas NEDP1 is the deneddylase. (**a**) HEK293T cells were transfected with His-NEDD8 and FLAG-PB2 along with Myc-XIAP, Myc-HDM2, Myc-TRIM40, Myc-Smurf1, Myc-RNF111 and HA-SCCRO, HA-RBX1 and HA-c-CBL expression plasmids, respectively. (**b**) HEK293T cells were transfected with His-NEDD8 and FLAG-PB2 along with Myc-HDM2 or Myc-HDM2 C464A expression plasmids. (**c**) HEK293T cells were transfected with FLAG-PB2, along with Myc-HDM2 or Myc-XIAP, and His-ubiquitin or His-NEDD8 expression plasmids. For (**a**), (**b**) and (**c**), cell lysates were harvested and subjected to His-pulldown assay, followed by immunoblotting with anti-FLAG antibody. Total cell lysates were immunoblotted with indicated antibodies. (**d**) HEK293T cells were co-transfected with Myc-HDM2 and FLAG-PB2 expression plasmids. The cell lysates were immunoprecipitated with anti-Myc agarose beads and immunoblotted with anti-FLAG antibody. (**e**) 293 T cells were transfected with control siRNA or HDM2 siRNA (#1 or #2) for 24 h and then transfected with FLAG-PB2 and His-NEDD8 expression plasmids for 36 h. Cell lysates were harvested and subjected to His-pulldown assay, followed by immunoblotting with anti-FLAG antibody. The total cell lysates were immunoblotted with indicated antibodies.

To identify the E3 ligase responsible for the NEDDylation of PB2, we transfected HEK293T cells with FLAG-PB2 and His-NEDD8 together with plasmids expressing one of the known E3 ligases (HDM2, XIAP, TRIM40, Smurf1, RNF111, c-CBL, SCCRO or RBX1)^30^, and performed the His-pulldown assay. The results showed that HDM2 can significantly enhance the intensity of PB2 NEDDylation (Fig. 2a). We then generated a HDM2 C464A mutant, which lacks E3 ligase activity^31^, and repeated the experiment. The data indicated that HDM2 C464A can no longer promote the NEDDylation of PB2 (Fig. 2b).

Since HDM2 is also known to be a ubiquitin E3 ligase, we wondered whether it specifically promotes PB2 NEDDylation. To address this question, we transfected HEK293T cells with plasmids expressing His-ubiquitin or His-NEDD8 together with HDM2 or XIAP as a negative control. The cell lysates were subjected to His-pulldown assay. As shown in Fig. 2c, HDM2 only promotes the NEDDylation, but not ubiquitination, of PB2. We next examined whether HDM2 can interact with PB2 by a co-immunoprecipitation assay. The data showed that HDM2 can bind to PB2 (Fig. 2d). To examine the effect of endogenous HDM2 on PB2 NEDDylation, we performed the HDM2 knockdown experiments. As shown in Fig. 2e, NEDDylation of PB2 was reduced in HEK293T cells treated with two sets of HDM2 siRNAs. These results demonstrated that HDM2 functions as an E3 ligase to mediate the NEDDylation of PB2.

### Overexpression of HDM2 and NEDD8 inhibits IAV replication

**Figure 3:**
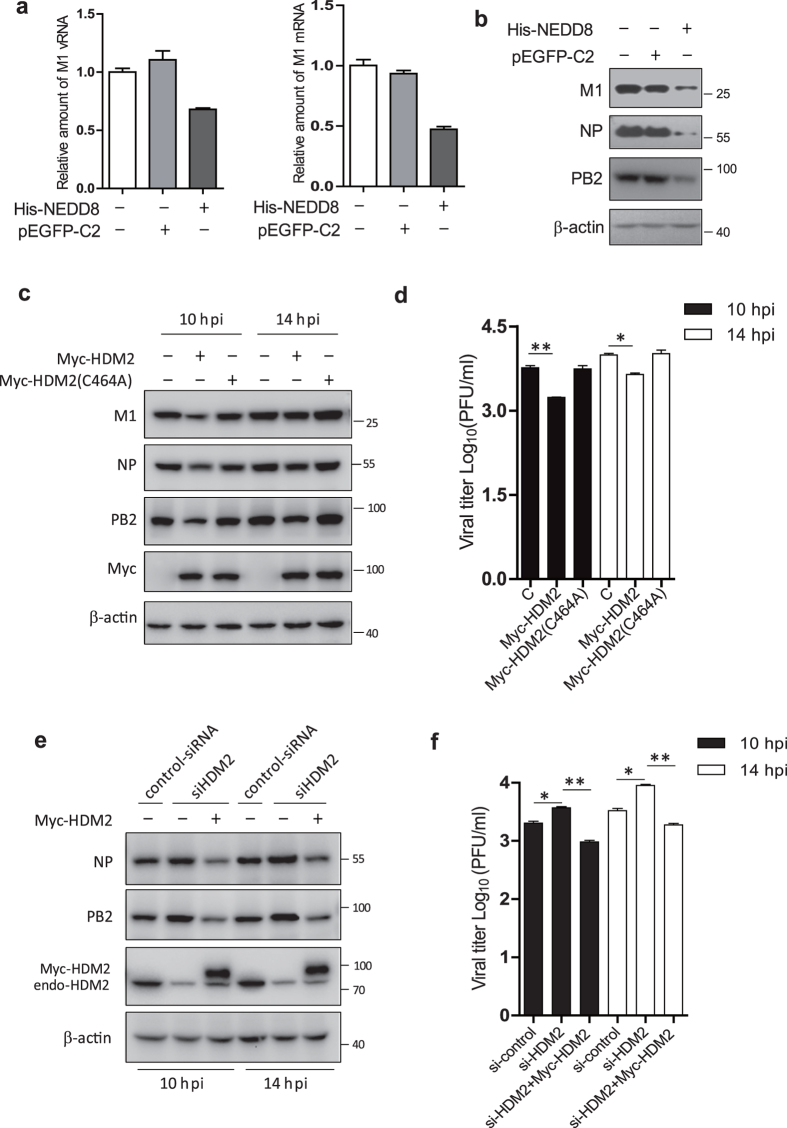
Overexpression of HDM2 and NEDD8 inhibits IAV replication. (**a**,**b**) HEK293T cells were transfected with pEF-His-NEDD8 and empty vector or pEGFP-C2 as control for 24 h and then infected with WSN at an MOI of 0.5. The cells were harvested at 6 h post-infection, and the total RNA was extracted and subjected to RT-qPCR with primers specific for M1 vRNA and mRNA (**a**), the cell lysates were subjected to immnoblotting with indicated antibodies (**b**). (**c**,**d**) HEK293T cells were transfected with pCMV-Myc-HDM2 or pCMV-Myc-HDM2 C464A, respectively. At 24 h post-transfection, the cells were infected with WSN virus at an MOI of 0.5 for 10 h and 14 h respectively. The cell lysates were harvested and subjected to immunoblotting with the indicated antibodies (**c**). The supernatants of infected cells were collected for plaques assay to measure the virus titer (**d**). (**e**,**f**) HEK293T cells were transfected with control siRNA or si-HDM2 for 24 h and then transfected pCMV-Myc-HDM2, and then infected with WSN at an MOI of 0.5 for 10 h and 14 h respectively. The cell lysates were harvested for immunoblotting with the indicated antibodies (**e**). The supernatants of infected cells were collected for plaque assay to measure the virus titer (**f**). The data of d and f were expressed as the mean of triplicated samples from 2 independent experiments. *p < 0.05, **p < 0.01.

To examine whether NEDDylation affects IAV replication, we transfected A549 cells with His-NEDD8 or pEGFP-C2 as a control, and infected the cells with IAV at an MOI of 0.5. The cells were harvested at 6 h post-infection, and then subjected to analysis by real-time PCR and immunoblotting. The levels of vRNA and mRNA of M1 were found to be reduced in NEDD8-overexpressing cells (Fig. 3a). The levels of M1, NP and PB2 proteins were also decreased in cells overexpressed NEDD8 compared to that in control cells (Fig. 3b). Since HDM2 is the E3 ligase for PB2 NEDDylation, we transfected A549 cells with pCMV Myc-HDM2 or control plasmids and then infected these cells with IAV (A/WSN/33 (H1N1)). Cell lysates were harvested for immunoblotting and supernatants were collected for plaque assay to determine virus titers. The immunoblotting data indicated that the amount of M1, NP and PB2 proteins were also reduced in HDM2 transfected cells compared to that in control cells (Fig. 3c), and virus titers were significantly lower in HDM2 overexpressed cells than that of control cells (Fig. 3d).

To examine the effect of endogenous HDM2 on viral replication, we performed the HDM2 knockdown and rescue experiments. We transfected A549 cells with si-HDM2 alone or together with pCMV-Myc-HDM2, and then infected the cells with IAV. The cell lysates were harvested for immunoblotting and supernatants were collected for plaque assay. The data showed that the protein levels of NP and PB2 (Fig. 3e) and viral titers (Fig. 3f) were higher in HDM2 knockdowned cells than that in control cells, while Myc-HDM2 can rescue the effect of HDM2 knockdown on viral replication. Taken together, these data suggested that HDM2 negatively regulates IAV replication.

### K699 is the major NEDDylation site in PB2

**Figure 4:**
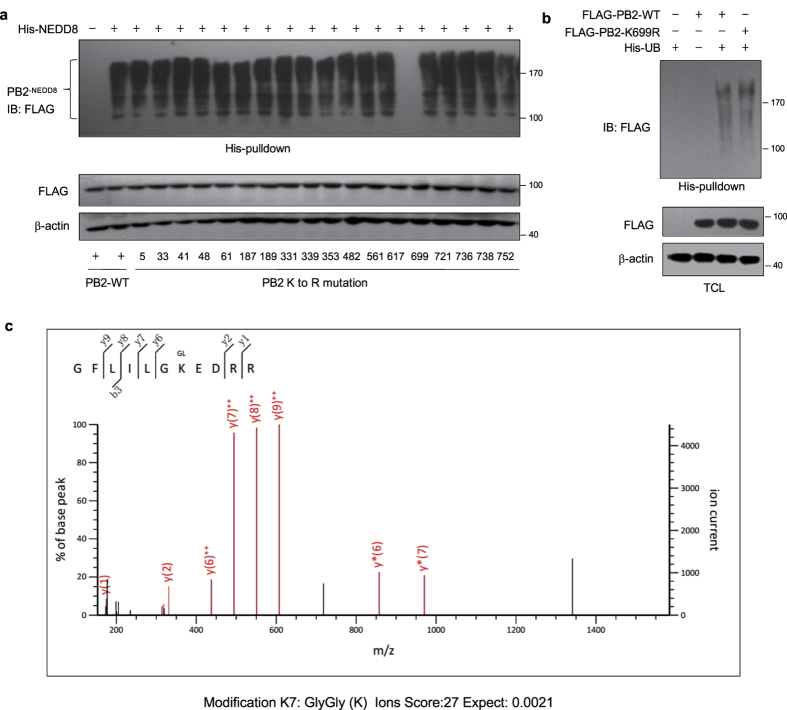
Residue K699 is the major NEDDylation site in PB2. (**a**) HEK293T cells were transfected with expression plasmids encoding FLAG-PB2 or FLAG-PB2 with K-to-R mutations at the indicated positions together with pEF-His-NEDD8 for 48 h. (**b**) HEK293T cells were transfected with FLAG-PB2 or FLAG-PB2 K699R and His-ubiquitin expressing plasmids. For (**a**) and (**b**), cell lysates were harvested and subjected to His-pulldown assay, followed by immunoblotting with anti-FLAG antibody. Cell lysates were immunoblotted with indicated antibodies. (**c**) HEK293T cells were transfected with pCMV-Myc-PB2 (aa 600–759) for 48 h. Cell lysates were immunoprecipitated with anti-Myc beads. Purified Myc-PB2 (aa 600–759) was subjected to mass spectrometry analysis. b- and y-ion designations are shown on the figure.

We next attempted to identify the NEDDylation site (s) in PB2. It is known that NEDDylation occurs on the lysine residues of target protein. By comparing the amino acid sequences of PB2 from eight different IAV strains (A/Mexico/4646/2009, A/New York/1/1918, A/Puerto Rico/8/1934, A/WSN/1933, A/Udorn/72, A/Japan/305/1957, A/Hong Kong/470/1997 and A/Hong Kong/1/1968), 18 conserved lysines were found. We generated PB2 mutants containing individual mutations of these lysines to arginines, and examined their NEDDylation levels in HEK293T cells. The level of NEDDylation in the K699R mutant was dramatically reduced compared to that of wild type and other PB2 mutants (Fig. 4a), which suggested that residue K699 is the major NEDDylation site. Since PB2 can also be modified by ubiquitin, we compared the level of ubiquitination of wild-type PB2 (PB2-WT) and mutant PB2 K699R (PB2-K699R). As shown in Fig. 4b, the level of ubiquitination of PB2-WT and PB2-K699R are comparable, suggesting that K699 is not a ubiquitination site. Furthermore, we performed mass- spectrometry (MS) analysis to determine whether PB2 K699 was modified. We transfected HEK293T cells with pCMV-Myc-PB2 (aa 600–759), together with plasmids expressing His-NEDD8. The Myc-PB2 (aa 600–759) was purified with anti-Myc agarose beads and subjected to MS analysis. The data indicated that the peptide GFLILGK (699) EDRR of PB2 was GlyGly-modified (Fig. 4c). Taken together, these data demonstrate that PB2 K699 is the major NEDDylation site in PB2.

### NEDDylation reduces PB2 stability

**Figure 5:**
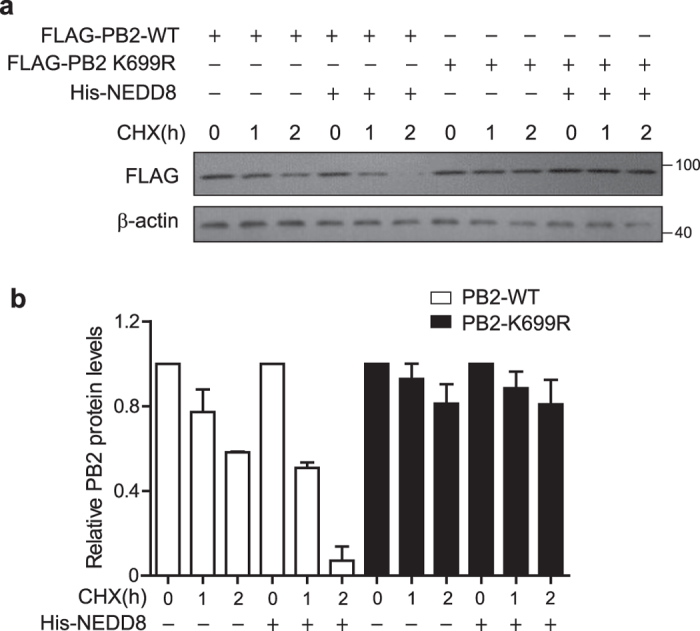
NEDDylation of PB2 reduced its stability. HEK293T cells were transfected with FLAG-PB2 or FLAG-PB2 K699R along with or without His-NEDD8 expression plasmids for 36 h. Cells were treated with 100 μg/ml of CHX at indicated times. The total cell lysates were harvested for immunoblotting with anti-FLAG antibody and anti-β-actin as control (**a**). Relative PB2 protein levels were quantified and the data represents the average of two independent experiments (**b**).

To examine whether NEDDylation of PB2 influences its stability, we compared the half-life of PB2-WT and PB2-K699R. We transfected HEK293T cells with pCMV-FLAG-PB2 or pCMV-FLAG-PB2 K699R with or without pEF-His-NEDD8, and then treated the cells with cycloheximide (CHX) for 1 and 2 h. The total cell lysates were harvested for immunoblotting to determine the amount of FLAG-PB2 and FLAG-PB2 K699R (Fig. 5a). The relative levels of FLAG-PB2 and FLAG-PB2 K699R from 2 independent experiments were quantified (Fig. 5b). The data indicated that the level of PB2-WT was decreased in cells treated with CHX and reduced more dramatically in NEDD8 overexpressed cells, whereas the level of PB2-K699R did not decrease even in cells overexpressing His-NEDD8.

### NEDDylation of PB2 hinders the replication of IAV

**Figure 6:**
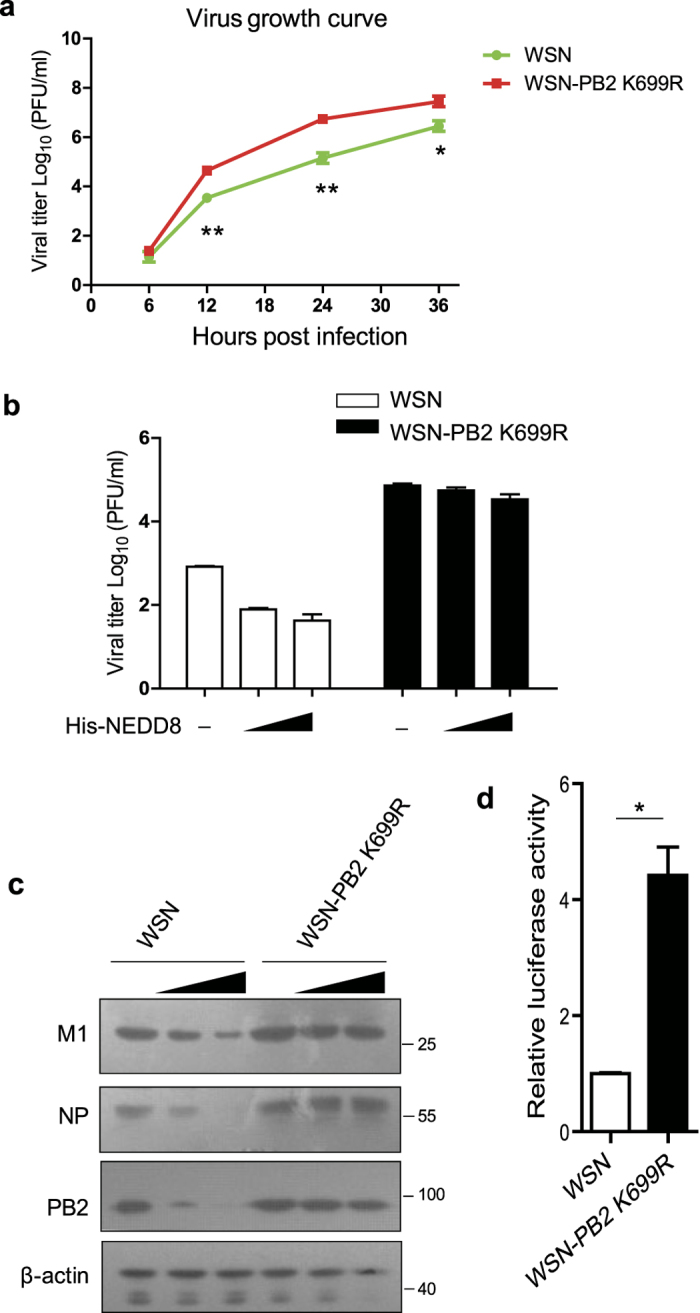
NEDDylation of PB2 hinders the replication of IAV. (**a**) MDCK cells were infected with WSN or WSN-PB2 K699R at an MOI of 0.01. The supernatants were harvested for plaque assay. (**b**,**c**) A549 cells were transfected with or without His-NEDD8 for 24 h and then infected with WSN or WSN-PB2 K699R at an MOI of 0.1 for 16 h. The supernatants were collected for plaque assay (**b**). The experiment is repeated twice and results were found to be comparable, while all samples were assayed in triplicate. The presented data is the mean from one experiment. Cell lysates were harvested and subjected to immunoblotting with indicated antibodies (**c**). (**d**) 293T-IAV-Luc cells were infected with WSN or WSN-PB2 K699R at an MOI of 0.1 for 12 h. The cell lysates were harvested for luciferase assay. The experiment was repeated twice and results were found to be consistent. The presented data is the mean of three biological samples from one experiment. *p < 0.05.

To understand the biological significance of NEDDylation of PB2 K699 in the propagation of IAV, we generated an IAV mutant in which PB2 was mutated to PB2 K699R (WSN-PB2 K699R) by taking the approach of reverse genetics. Then we compared the proliferation kinetics of WSN with WSN-PB2 K699R. We infected MDCK cells with WSN or WSN-PB2 K699R and harvested the supernatants at different time points (6, 12, 24, and 36 h post-infection) for plaque assay. WSN-PB2 K699R replicated much more efficiently than WSN in MDCK cells, resulting in higher virus titers. At 12 h, 24 h and 36 h post infection, the titers of WSN-PB2 K699R were significantly higher than that of WSN (Fig. 6a), This result suggested that NEDDylation of PB2 K699 restricted optimal virus growth.

Furthermore, we examined the replication of wild type WSN virus and WSN-PB2 K699R mutant in A549 cells transfected with NEDD8 expressing plasmid or control plasmid. We analyzed virus titers in the supernatant by plaque assay and compared protein levels of M1, PB2 and NP by immunoblotting. The data showed that the titers of WSN were lower than that of WSN-PB2 K699R. More importantly, we found that the titers of WSN were significantly reduced in the NEDD8-overexpressing cells while the titers of WSN-PB2 K699R were comparable between control and NEDD8-overexpressing cells (Fig. 6b). Consistently, immunoblotting data indicated that the protein levels of M1, NP and PB2 of WSN but not WSN-PB2 K699R in NEDD8- overexpressing cells were greatly decreased compared to that in control cells (Fig. 6c). Furthermore, we generated the vRNP reporter 293 T cells (293T-IAV-Luc), in which the luciferase gene driven by NP promoter was integrated in the genome of cells. We infected the 293T-IAV-Luc cells with WSN or WSN-PB2 K699R and harvested the cell lysates for luciferase assay. The data showed that luciferase activity in WSN infected cells were much lower than that in WSN-PB2 K699R infected cells (Fig. 6d). These results strongly suggested that NEDDylation of PB2 at K699 suppressed the replication of IAV.

### WSN-PB2 K699R virus possesses higher virulence than WSN

**Figure 7:**
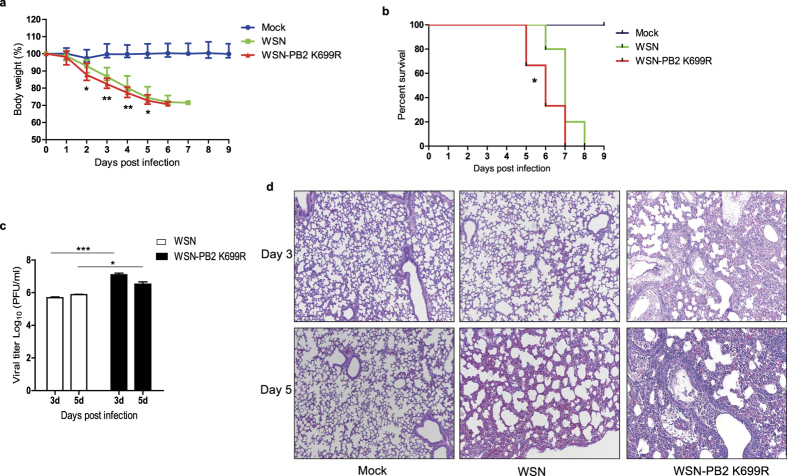
WSN-PB2 K699R possesses higher virulence than WSN. (**a**,**b**,**c**,**d**) BALB/c mice (7 weeks old, female) were infected intranasally with PBS (n = 10), WSN (n = 10) or WSN-PB2 K699R (n = 9), respectively (10000 PFU each). The body weight (**a**) and survival (**b**) of mice were monitored daily. At 3 and 5 dpi, the lungs of infected mice (n = 3) were collected to measure the virus titer (**c**) and as well as histopathological analysis (**d**). Representative histological images of lung tissues from mock-challenged mice (left panels), mice treated with WSN (middle panels) or WSN-PB2 K699R (right panels) were shown. All Experiments for the plague assay were repeated twice and results were found to be comparable. The presented data were expressed as the mean from one experiment. *p < 0.05; **p < 0.01; ***p < 0.001.

We next assessed the impact of NEDDylation of PB2 on virus pathogenicity in mice. We infected BALB/c mice intranasally with WSN or WSN-PB2 K699R at a dose of 10000 PFU, measuring the body weights and monitoring for survival daily after challenge. Mice infected with WSN-PB2 K699R exhibited more severe weight loss compared to those infected with WSN (Fig. 7a). Also, WSN-PB2 K699R caused 30% mortality as early as at 5 day post-infection (dpi), and 67% mortality at 6 dpi, which were higher than that caused by WSN (Fig. 7b). We also examined the viral titers in the lungs of infected mice at both 3 and 5 dpi. Consistently, viral titers in lungs of mice infected with WSN-PB2 K699R were higher than that of WSN infected mice at both time points (Fig. 7c). In addition, we performed histological analysis of the lungs from infected mice at 3 and 5 dpi. Both viruses caused bronchus-alveolar epithelial degeneration and interstitial pneumonia, as well as focal or diffuse lung damage. The peribronchiolar and perivascular areas of infected mice were infiltrated by numbers of lymphocytes and plasma cells. However, the alveolar damage and interstitial inflammatory infiltration in WSN-PB2 K699R mice were much more severe than that of WSN infected mice. Moreover, necrosis was observed in the lung tissues of mice infected with WSN-PB2 K699R (Fig. 7d). These results illustrate that WSN-PB2 K699R causes more severe pathogenicity in mice than that of WSN, suggesting that preventing NEDDylation of PB2 promotes virus propagation and enhances virulence.

## Discussion

The NEDD8 protein is a type of ubiquitin-like protein (UBL) and shares the greatest similarity to ubiquitin among all of the UBLs, and can be conjugated to substrates in a process known as NEDDylation^21^. NEDDylation plays pivotal roles in regulating cellular processes as well as the viral life cycle^32^. Ubiquitin and other ubiquitin-like proteins, such as SUMO and ISG15, have been found to regulate the IAV life cycle^16^,^33^,^34^,^35^. However, whether NEDDylation modification is involved in the replication of IAV is not known. In this study, we demonstrated that PB2 can be modified by NEDDylation, which reduces its stability and inhibits the replication of IAV *in vitro* and *in vivo*.

PB2 is an 87-kDa basic cap-binding protein involved in the initiation of viral transcription as well as viral replication. The N- and C-terminal regions of PB2 contain independent NP binding sites, located between residues 1–269 and 580–683, respectively. The interaction of PB2 with NP affected the activity of RNPs, and may be involved in regulating the switch from viral transcription to replication^36^,^37^,^38^. Although we did not observe that PB2 NEDDylation influences the association of PB2 with NP, we cannot rule out the possibility that NEDDylation of PB2 could affect the formation of vRNP complexes. It has been reported that two regions of PB2 (aa 448–496 and aa 736–739) were required for the nuclear localization of influenza virus PB2^39^,^40^. It will be interesting to further analyze whether PB2 NEDDylation affects its subcellular localization in addition to influence its stability.

Recently, the crystal structures of influenza viral polymerase were solved^41^,^42^. PB2 K699 is exposed at the external loop of the trimeric viral polymerase, and is likely to be modified by NEDD8. According to the structure of PB2 and other viral polymerase subunits, the PB2 K699R mutation would not affect the tertiary structure of PB2 or the trimeric viral polymerase. However, the NEDDylation of PB2 K699 may influence the structure of PB2 and consequently interfere with the function of vRNP.

It has been reported that some mutated influenza viruses grow faster *in vitro* than wild type viruses, but peak lung viral titers *in vivo* were not observed to be different among these viruses^43^. We compared WSN and WSN-PB2 K699R growth kinetics in cell culture, in addition to virulence in a mouse model. We observed that WSN-PB2 K699R replicated better than WSN in MDCK cells, and consistently WSN-PB2 K699R causes more severe pulmonary damage and results in higher lethality rates. In addition, the viral titers in the lungs of mice infected with WSN-PB2 K699R were higher than animals in the WSN group. Our results demonstrate that NEDDylation of PB2 influences viral replication both *in vitro* and *in vivo*. In order to know whether PB2 K699 is conserved or not among the different influenza virus strains, we compared the sequences of 3500 publically-available influenza virus strains in the NCBI database (http://www.uniprot.org/uniprot/?query=pb2+AND+influenza+virus&sort=score). The data showed that PB2 K699 is highly conserved and present in 3399 out of 3500 strains, and 95 strains are with PB2 R699, indicating that the virus strains with PB2 K699 are predominant. We postulate that the virus with PB2 K699R mutation may proliferate better in cultured cells due to avoiding been NEDDylated, but on the other hand it may not adapt to host as well as the strain with PB2 K699 which may limit it to spread out. However this assumption needs further investigated.

Taken together, this study revealed that NEDDylation of PB2 by HDM2 negatively regulates influenza A viral replication. Our results suggested that NEDDylation modification on viral protein could be a host antiviral manner. These findings will contribute to our understanding of NEDDylation and its role in regulating viral infection.

## Methods

### Cell Culture, Virus Strains and Reagents

Human embryonic kidney HEK293T cells (HEK293T), Madin-Darby canine kidney cells (MDCK), and human lung adenocarcinoma epithelial cells (A549) were obtained from China Infrastructure of Cell Line Resources and maintained in Dulbecco’s modified Eagle’s medium (DMEM) (Invitrogen) supplemented with 10% heat-inactivated fetal bovine serum (FBS) and antibiotics (100 U/ml penicillin G and 100 g/ml streptomycin).

The A/WSN/33 (H1N1) (WSN) strain of IAV were grown in MDCK cells.

Anti-M1 monoclonal antibody and anti-NP polyclonal antibody were gifts from Prof. Wenjun Liu. Anti-Myc (9E10) and FLAG (M2) antibodies were purchased commercially (Sigma). Anti-His (H-3) and anti-β-actin (1–19) antibodies were also purchased commercially (Santa Cruz Biotechnology). Anti-NEDD8 (Y297) was purchased commercially (Abcam).

### Plasmid Construction

The NEDD8, ubiquitin and SUMO1 genes were cloned into pEF1/His (Invitrogen). Plasmids pHH21-PB2 K699R and pCAGGS-PB2 K699R for generating mutant WSN-PB2 K669R virus were created by site specific mutagenesis with FastPfu DNA Polymerase (TransGen Biotech). All cloned cDNAs and mutations were confirmed by DNA sequencing. PB2 genes from various IAV subtypes (H3N2, H7N9, H9N2) were kindly provided by Honglin Chen from the University of Hong Kong. The plasmids used in IAV reverse genetics were provided by Yoshihiro Kawaoka (University of Wisconsin School of Veterinary Medicine)^44^. HA-SCCRO expressing plasmid was a gift from Bhuvanesh Singh. Myc-Smurf1 expressiong plasmid was provided by Lingqiang Zhang (Academy of Military Medical Sciences). Myc-HDM2, Myc-XIAP, Myc-RNF111 and Myc-TRIM40 expressing plasmids were gifts from Jianping Jin (University of Texas Medical School). The IAV polymerase reporter plasmid pREP4-FluA-Luc was provided by Andrew Pekosz (Johns Hopkins University)^45^. Briefly, the DNA fragment containing RNA polymerase I promoter-NP 5′UTR- luciferease-NP 3′UTR-RNA polymerase I terminator was cloned into the *PvuII* site of pREP4 and named pREP4-FluA-Luc.

### RNA Interference

A549 cells were transfected with HDM2-specific small interfering RNAs (siRNAs) targeting 3′ UTR of HDM2 mRNA or negative control siRNA (100 nM) (Genepharma) using Lipofectamine 2000 (Invitrogen) according to the manufacturer instructions. The sequences of siRNAs are as follows: 5′-UUCUCCGAACGUGU CACGU-3′(control-siRNA); 5′-CCUUUACACCAACUCCUAA-3′ (siHDM2#1); 5′-GUC CAGCCAAGAAUUAGUA-3′ (siHDM2#2).

### His-pulldown Assay

His-pulldown assays were performed to examine the post-translational modification of proteins. Briefly, HEK293T cells in 10 cm dish were transfected with pEF-His-NEDD8 and the indicated plasmids for 48 h. Cells were harvested and lysed in denaturing lysis buffer (6 M guanidinium-HCl, 0.1 M Na_2_HPO_4_ and NaH_2_PO_4_, 0.01 M Tris-HCl, pH 8.0, 5 mM imidazole and 10 mM β-mercaptoethanol) for 30 min at room temperature. 70 μl of Ni^+^ beads were added to the lysate for 4 h and then washed with washing buffer. The samples were eluted with 30 μl of elution buffer (0.15 M Tris-HCl, pH 6.7, 30% Glycerol, 5% SDS, 500 mM imidazole) and analyzed by SDS-PAGE and immunoblotting.

### Immunoprecipitation

HEK293T cells in 10-cm dishes were transfected with the indicated plasmids for 48 h. Cells were collected and lysed in RIPA lysis buffer (50 mM Tris-HCl, pH 7.4, 150 mM NaCl, 1% NP-40, 1 mM EDTA) supplemented with protease inhibitor (Roche) for 15 min at 4 °C, and then centrifuged at 13400 × g for 25 min. The supernatants were incubated with 10 μl of the indicated antibody agarose slurry (Sigma) for 4 h at 4 °C, and then washed three times with lysis buffer. The samples were dissolved in SDS loading buffer and subjected to immunoblotting.

### Generation of Recombinant Viruses

The reverse-genetics systems for the generation of recombinant IAV (WSN) have been described previously^44^,^46^. For the generation of recombinant WSN viruses, HEK293T cells were cotransfected with eight viral genome-expressing plasmids (pHH21 PB2 for wild type WSN virus, or pHH21 PB2-K699R for WSN-PB2 K699R virus) and four pCAGGS protein expression plasmids encoding the viral polymerase subunits PB1, PB2 and PA (pCAGGS PB2 for wild type WSN virus, or pCAGGS PB2-K699R for WSN-PB2 K699R virus) as well as NP. At 48 h post-transfection, recombinant viruses were passaged to fresh MDCK cells for virus amplification and rescue.

### Viral Growth Kinetics

A549 cells were infected with wild-type A/WSN/33 (H1N1) or mutant virus WSN-PB2 K699R at an MOI of 0.01. At 1 h post infection, the cells were cultured in serum-free medium. The supernatants were harvested at the indicated times, and virus titers were determined by plaque assay on MDCK cells.

### Plaque Assay

MDCK monolayer cells in 12-well dishes were washed twice with PBS, followed by infection with serial 10-fold dilutions of virus in serum-free DMEM medium supplemented with 4 μg/ml of TPCK-trypsin (Sigma), and incubated at 37 °C for 1 h. Afterwards, the cells were washed with PBS and overlaid with Modified Eagle’s Medium (MEM) containing 1% agarose (AMRESCO) and 2 μg/ml of TPCK-trypsin. After 2 days, visible plaques were counted and viral titers were calculated.

### Generation of 293T-IAV-Luc Cell Line

HEK293T cells were transfected with NP promoter reporter plasmid pREP4-FluA-Luc for 24 h. The cells were then selected with 200 μg/ml hygromycin for 2 weeks. Survived cells were maintained in 100 μg/ml hygromycin and named 293T-IAV-Luc.

### Anhimal Model and Virus Challenge

Female seven-week old BALB/c mice were obtained commercially (Vital River Laboratories). The animals were kept in special pathogen free (SPF) facilities in biosafety level-2 housing. Infection was performed by intranasal inoculation of mice anesthetized by diethyl ether.

### Ethics Statement

Mice studies were reviewed and approved by the Ethics Committee of Animal Experiment and Human Medical Research of the Institute of Microbiology (approval no. APIMCAS2015027). All animal experiments were performed in accordance with relevant guidelines and regulations.

### Lung Histopathology

The mice were infected with virus at the indicated times. Lungs harvested from mice were fixed in 10% buffered formalin and embedded in paraffin wax. Sections with 5 μm in thickness were mounted on slides for haematoxylin and eosin (H&E) staining.

### Mass Spectrometry

HEK293T cells were transfected with Myc-PB2 and His-NEDD8 for 48 h. Cells were then collected and lysed in RIPA lysis buffer for 15 min at 4 °C, and then centrifuged at 13400 × g for 25 min. The supernatants were incubated with 10 μl of Myc antibody agarose slurry (Sigma) for 4 h at 4 °C, and then washed with lysis buffer three times. Myc-PB2 were removed from the agarose beads by competition with Myc-peptide (Sigma) and then trypsinized. The digested peptides were analyzed by HPLC-ESI/MS/MS.

### Real-time PCR

Total RNA was extracted from cells using TRIzol reagent (Invitrogen). First-strand cDNA was synthesized by the reverse transcription using sense-specific primers for vRNA (5′-GCTGCAATGACGAGAGGATC-3′) and oligo (dT) for mRNA. Quantitative PCR was performed in 25 μl reactions using SYBR Green PCR master mix (TOYOBO). All data were normalized to β-actin. Primer sequences for specific genes are as follows: β-actin, forward, 5′-GTGAAGGTGACAGCAGTCGGTT-3′ and reverse, 5′-GAAGT GGGGTGGCTTTTAGGA-3′; M1, forward 5′-ACTTGAATCGTTGCATCTGC-3′ and reverse 5′-GAGGCCATGGATATTGCTAG-3′.

### Statistical Analysis

Statistical comparisons were performed using GraphPad Prism version 5.0 (GraphPad software Inc.). Student’s t-test was used to analyze the data. Log-rank (Mantel-Cox) test was used to compare the survival curves. The differences between the variants were considered to be statistically significant if p < 0.05, and very significant if p < 0.01. Error bars represent standard error (±SEM).

## Additional Information

**How to cite this article**: Zhang, T. *et al*. NEDDylation of PB2 Reduces Its Stability and Blocks the Replication of Influenza A Virus. *Sci. Rep.* **7**, 43691; doi: 10.1038/srep43691 (2017).

**Publisher's note:** Springer Nature remains neutral with regard to jurisdictional claims in published maps and institutional affiliations.
